# BMS‐202, a PD‐1/PD‐L1 inhibitor, decelerates the pro‑fibrotic effects of fibroblasts derived from scar tissues via ERK and TGFβ1/Smad signaling pathways

**DOI:** 10.1002/iid3.693

**Published:** 2022-09-07

**Authors:** Yuanyuan Cai, Min Xiao, Xinqing Li, Shanyu Zhou, Yangyang Sun, Wenyuan Yu, Tianlan Zhao

**Affiliations:** ^1^ Department of Plastic and Cosmetic Surgery The Second Affiliated Hospital of Soochow University Soochow Jiangsu China; ^2^ Department of Plastic and Cosmetic Surgery Changzhou No.2 People's Hospital Changzhou Jiangsu China; ^3^ Department of Oncology Changzhou Cancer Hospital Affiliated to Soochow University Changzhou Jiangsu China; ^4^ Department of Pathology Changzhou No.2 People's Hospital Changzhou Jiangsu China

**Keywords:** BMS‐202, fibroblasts, hypertrophic scar, PD‐1/PD‐L1 inhibitor

## Abstract

**Introduction:**

Hypertrophic scar (HS), a fibroproliferative disorder of the skin with some tumor‐like properties, is closely related to dysregulated inflammation. PD‐1/PD‐L1 inhibitor is a promising medication for cancer therapy as its potent functions on adaptive immune response; whether it could be a candidate for HS therapy has aroused our interest. This study aimed to explore the effect and the mechanism of BMS‐202, a PD‐1/PD‐L1 inhibitor, in HS.

**Methods:**

Ten HS and adjacent normal skin tissues collected from HS patients were used to detect α‐SMA, collagen I, and PD‐L1 expression by Quantitative reverse transcription‐polymerase chain reaction and western blot (WB) analysis. Fibroblasts derived from HS tissues (HFBs) were exposed to diverse concentrations of BMS‐202, of which proliferation, migration, apoptosis, and collagen synthesis were evaluated by Cell Counting Kit‐8, wound healing, terminal deoxynucleotidyl transferase (TdT) dUTP Nick‐End labeling, and [^3^H]‑proline incorporation assays, respectively. The effect of BMS‐202 on α‐SMA and collagen I expression, and transforming growth factor beta 1 (TGFβ1)/Smad signaling in HFBs was also determined by WB and enzyme‐linked immunosorbent assay.

**Results:**

The expression level of PD‐L1 was significantly elevated in both HS tissues and HFBs, which was positively correlated with α‐SMA and collagen I expressions. BMS‐202 exerted a significant suppression effect on the cell proliferation, migration, collagen synthesis, and α‐SMA and collagen I expression of HFBs in a concentration‐dependent way; but did not affect apoptosis. Finally, BMS‐202 could reduce the phosphorylation of ERK1/2, Smad2, and Smad3, and the TGFβ1 expression once its concentration reached 2.5 nM.

**Conclusion:**

BMS‐202 effectively suppressed proliferation, migration, and extracellular matrix deposition of HFBs, potentially through the regulation of the ERK and TGFβ1/Smad signaling pathways.

## Graphical abstract

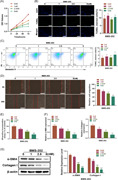

## 1 INTRODUCTION

Hypertrophic scar (HS) is caused by an abnormal response to wound, which is characterized by fibroblast hyper‐proliferation with the excessive deposition of extracellular matrix (ECM) proteins. It has been reported that the incidence of HS ranges from 32% to 94% following injuries.^1^ Even though not life‐threatening, HS often causes cosmetic and functional impairments for patients, thereby causing an adverse impact on patients' quality of life. For treating HS, there are many strategies, which include local corticosteroid injection, surgical excision, cryotherapy, and radiotherapy.^2^ However, many times, the most of above‐mentioned HS therapeutic strategies will cause several undesirable, even severe side effects. For example, corticosteroid injection might lead to pigmentation, skin atrophy, and increasing risks of dehiscence.^3^ Although surgical excision can quickly relieve symptoms, it is often accompanied by a high recurrence rate, which limits its application.^4^ Radiotherapy causes growth suppression and increases the risks of carcinogenesis.^5^ Therefore, the exploration of novel HS therapy is still an urgent need.

Like other types of tissue, the repair and regeneration of skin tissues is a complex process involving the inflammatory microenvironment.^6^ As known, inflammation is an indispensable defense mechanism for the body to resist tissue damage or pathogens.^7^ In the early stage of acute inflammation, the innate immune response recruits important inflammatory cells to start tissue repair; the second key stage is to reduce the proinflammatory response by converting proinflammatory macrophages into repair macrophages; in the final stage, inflammatory cells disappear from the injury site or eliminated through apoptosis to restore tissue homeostasis. However, persistent chronic inflammation usually impairs the repair/regeneration process and leads to scar formation.^8^ Hence, immune response plays an important role in HS formation. The better healing capacity of the oral mucosa has been explained by a diminished immune reaction when wounding occurs due to the less immune cell in the healthy oral mucosa.^9^ HS has often considered a kind of benign skin tumor since its main characteristic is the excessive proliferation of fibroblasts. One of the major players in creating HS in general is the myofibroblast, the predominant phenotype of cancer‐associated fibroblasts.^10^ Besides, the key regulatory factor of myofibroblast (transforming growth factor beta 1 [TGFβ1]) has substantial influence on HS formation, which also has long been recognized as a key molecule implied in tumor development and progression.^11^ Moreover, ECM remodeling, another main pathological feature of HS, has been implicated in tumor malignancy and metastatic progression.^12^ Over past decades, immunotherapy has been considered as one of the promising therapeutic tools for multiple types of solid tumors.^13^ Immune checkpoints, including PD‐1/PD‐L1 and CTLA‐4, are the most widely studied therapeutic targets for immunotherapy.^14^ In combination with the recent notion that HS shares several characteristics with cancer, we speculated that immune checkpoint inhibitors could also be a promising therapeutic agent for HS.

BMS‐202 is one of the entirely nonpeptidic organic BMS‐series of compounds disclosed by Bristol Myers Squibb, which has been proven to suppress the PD‐1/PD‐L1 interaction by inducing PD‐L1 dimerization.^15^ Several experimental studies on animals demonstrated that BMS‐202 was useful to prevent tumor metastasis and relapse in breast and pancreatic cancers.^16^, ^17^ Nevertheless, its role in HS formation has been hardly reported. This study aims to investigate the effect and potential mechanism of the PD‐1/PD‐L1 binding inhibitor BMS‐202 on the cell viability, migration, apoptosis, and the synthesis of collagen in fibroblasts derived from HS tissues (HFBs) to determine its potential for therapeutic applications for HS.

## 2 MATERIALS AND METHODS

### 2.1 **S**amples collection and cell isolation

Ten HS tissues and the adjacent normal skin (NS) tissues were collected from HS patients during scar surgical excision at the Second Affiliated Hospital of Soochow University. All enrolled patients signed written informed consent and did not receive any other therapy before surgery. This study was reviewed and approved by the Ethics Committee of the Second Affiliated Hospital of Soochow University (Approval Number KY115‐01).

Primary fibroblasts were isolated from the harvested HS and NS tissues as previously described.^18^ Briefly, collected tissues were subjected to removing excessive adipose tissues, followed by digested with Dispase II (Cat. no. D4693; Sigma‐Aldrich) to obtain dermis. Next, dermis tissues were washed in phosphate‐buffered saline (PBS) with gentle shaking overnight. Finally, the tissues were chopped and incubated with type I collagenase (Cat. no. SCR103; Sigma‐Aldrich) for 3 h to obtain HFBs or NFBs. The isolated cells were maintained in Dulbecco's modified Eagle medium containing 10% fetal bovine serum and 1% penicillin‐streptomycin at 37°C in a 5% CO_2_ incubator.

### 2.2 Quantitative reverse transcription‐polymerase chain reaction (qRT‐PCR)

Total RNA isolated from dermal tissues and fibroblasts using TRIzol reagent was obtained to generate complementary DNA (cDNA) for RT‐qPCR analysis. The amplification of cDNA was performed using QuantiTect PCR Kits (Qiagen) with 7500 Real‐Time PCR System (Applied Biosystems). The primer sequences used in this study were listed as followed: α‐SMA (forward: 5′‐AGCAGGCCAAGGGGCTATATAA‐3′; reverse: 5′‐TTCGTAGCTGTCTTTTTGTCCCA‐3′), Collagen I (forward: 5′‐GAACCTGGGATAGCAGGACAC‐3′; reverse: 5′‐CATAGTGGGTCCACAAAGACATC‐3′), PD‐L1 (Forward: 5′‐GGTGCCGACTACAAGCGAAT‐3′; reverse: 5′‐TAGCCCTCAGCCTGACATGTC‐3′), and β‐actin (forward: 5′‐CACCATTGGCAATGAGCGGTTC‐3′; reverse: 5′‐ AGGTCTTTGCGGATGTCCACGT‐3′). The relative expression of RNA was calculated using the 2^−ΔΔCt^ method.^19^

### 2.3 Western blot

Total protein was isolated from dermal tissues and fibroblasts by using RIPA buffer (10X) (Cell Signaling) and quantified by a BCA assay kit (Sigma‐Aldrich). After separating the total protein via sodium dodecyl sulfate–polyacrylamide gel electrophoresis gel, proteins were transferred onto polyvinylidene difluoride membranes. Then, membranes were blocked for 2 h and subsequently incubated with primary antibodies overnight. The next day, membranes were rinsed three times and further incubated with horseradish peroxidase‐conjugated secondary antibody for 2 h. Finally, protein bands were visualized with an ECL detection reagent (Sigma‐Aldrich). Antibodies used in this study were all supplied by Abcam, and the corresponding information was listed as followed: α‐SMA (Cat. no. ab5894; 1 µg/ml), collagen I (Cat. no. ab138492; 1/2000 dilution), phosphor‐ERK1/2 (p‐ERK1/2; Cat. no. ab278538; 0.1 µg/ml), ERK1/2 (T‐ERK1/2; Cat. no. ab184699; 1/10,000 dilution), phosphor‐Smad2 (p‐Smad2 (S467); Cat. no. ab280888; 1/1000 dilution), Smad2 (T‐Smad2; Cat. no. ab40855; 1/5000 dilution), phosphor‐Smad3 (p‐Smad3 (S423 + S425); Cat. no. ab52903; 1/2000 dilution), Smad3 (T‐Smad3; Cat. no. ab40854; 1/5000 dilution), and β‐actin (Cat. no. ab8226; 1 µg/ml).

### 2.4 Cell viability analysis

Cell Counting Kit‐8 (CCK‐8; Dojindo) was exploited to assess the effect of BMS‐202 on the cell viability of HFBs. In brief, cells were respectively treated with 0, 1, 2.5, and 5 nM of BMS‐202 for 24 h. After 24 h cultivation, the medium was replaced with 10% CCK‐8 solution in each well, and the plates were incubated for another 2 h. Finally, the absorbance at 450 nm of each well was detected using a microplate reader (BioTek).

### 2.5 Cell apoptosis assay

To examine the apoptosis of HFBs, terminal deoxynucleotidyl transferase (TdT) dUTP Nick‐End labeling (TUNEL) assay was performed as Kyrylkova et al.^20^ reported. In brief, HFBs were allowed to grow on chamber slides. After 24 h treatment, the cells were fixed with 4% paraformaldehyde, subsequently rinsed three times with PBS. DNA fragmentation was stained with green using DeadEnd™ Fluorometric TUNEL System (Promega) as per the manufacturer's instructions. Afterward, the TUNEL‐positive cells were counterstained with DAPI before observing and imaging under a microscope (at least six fields per sample).

The apoptosis rate of HFBs was also detected by Annexin‐V/PI staining and subsequent flow cytometry analysis. The HFBs with different treatments were harvested using Trypsin/ethylenediaminetetraacetic acid. After washing three times, the collected HFBs were stained with annexin V‐FITC and PI in the dark for 15 min. Finally, the percentage of Annexin‐V+/PI+ was determined on flow cytometry (BD Biosciences).

### 2.6 Cell migration analysis

The effect of BMS‐202 on the migration ability of HFBs was detected by performing wound healing assay. Briefly, cells were seeded into six‐well plates and cultured until a monolayer of cells had formed. The 200‐μl pipette tip was used to generate the similar size of scratches in the cell layer for each group. Next, scratched cells were removed; the remaining cells were treated with BMS‐202 and continually cultured for 24 h. After scratching for 0 and 24 h, the wound of each well was photographed with a microscope; and the migrated area was calculated.

### 2.7 Collagen quantification

[^3^H]‑proline incorporation assay was performed to investigate the effect of different doses of BMS‐202 on the collagen synthesis of HFBs. HFBs were plated in 24‐well plates at 8 × 10^4^ cells/well for 24 h treatment. Next, the cells were incubated with 0.5 μCi of [^3^H]‑proline for an additional 24 h; subsequently rinsed with PBS three times. Finally, exogenous ^3^H‐proline incorporation was determined in scintillation counter (Beckman). The results were represented as count per minute/cells.

### 2.8 Enzyme‐linked immunosorbent assay (ELISA)

By using commercial ELISA kit for human TGFβ1 (Cat. no. ab100647; Abcam), the concentrations of TGFβ1 were detected in the cell supernatants of diverse groups.

### 2.9 Statistics

All statistical analysis was conducted on GraphPad Prism 8.0.1 software. Data were expressed as the mean ± *SD*, and analyzed using the student *t* test or one‐way analysis of variance followed by Tukey's post hoc test. Pearson's correlation analysis was performed to analyze the correlation between the expression of PD‐L1 with α‐SMA and Collagen I. A *p* < .05 means that the difference was statistically significant.

## 3 RESULTS AND DISCUSSION

### 3.1 PD‐L1 is highly expressed and positively correlated with the expression of α‐SMA and Collagen I in HS tissues

**Figure 1:**
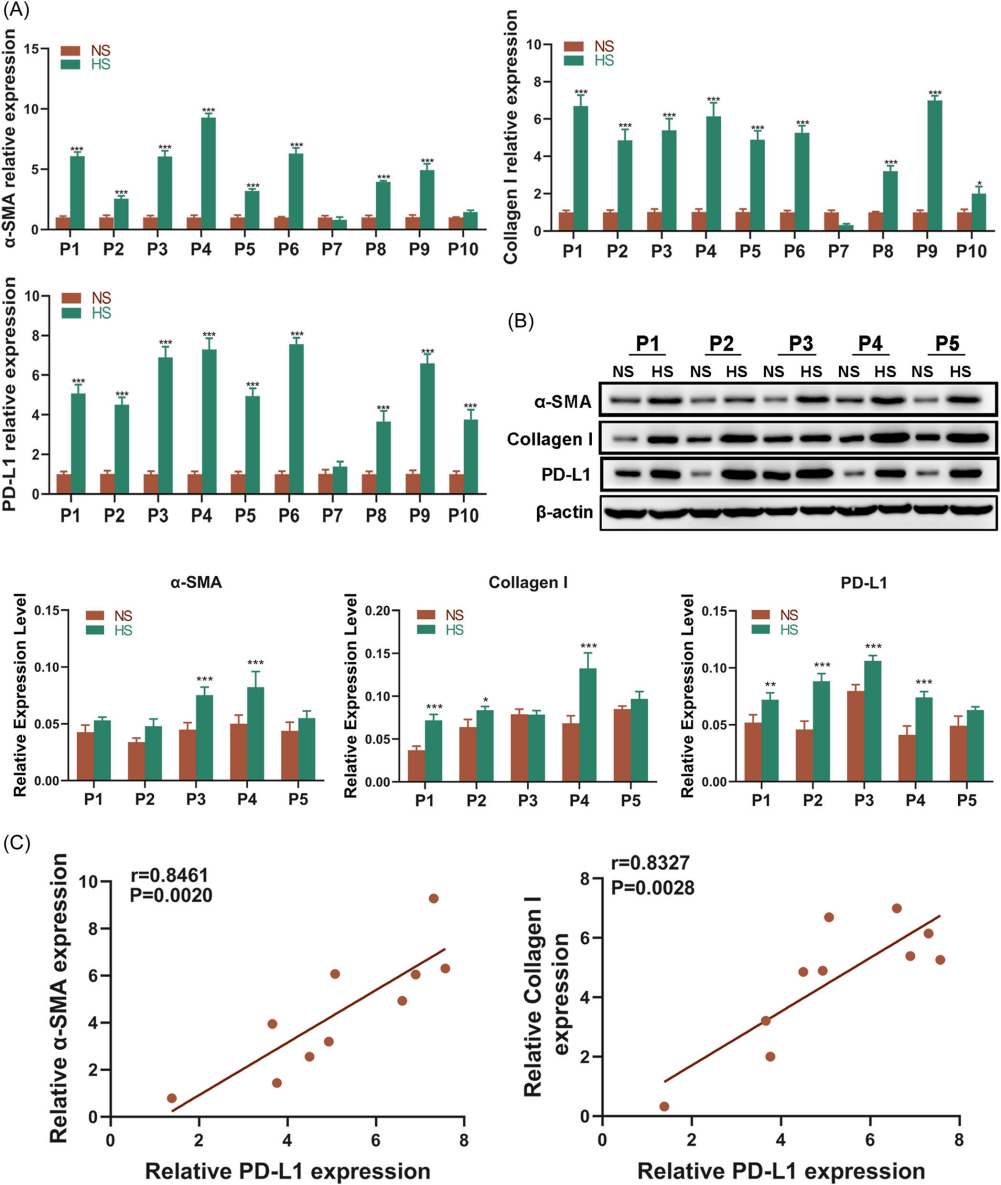
PD‐L1 is highly expressed and positively correlated with the expression of α‐SMA and Collagen I in HS tissues. (A) The mRNA expression level of PD‐L1, α‐SMA, and Collagen I between NS and HS tissues isolated from 10 patients was analyzed by qRT‐PCR. (B) The protein expression level of PD‐L1, α‐SMA, and Collagen I between NS and HS tissues isolated from five patients was analyzed by western blot. (C) The association between PD‐L1 with α‐SMA and Collagen I mRNA expressions in HS tissues was assessed by Pearson's correlation coefficient (*n* = 10). (*p < .05, **p < .01, and **p < .005 vs. the NS group). HS, hypertrophic scar; mRNA, messenger RNA; NS, normal skin; qRT‐PCR, quantitative reverse transcription‐polymerase chain reaction

To explore the potential role of PD‐L1 in HS, its expression between HS and NS tissues were analyzed. The qRT‐PCR analysis showed that the expression of PD‐L1 in HS tissues was significantly higher than that in NS tissues (Figure 1A), which as in line with the result of western blot (Figure 1B). It's widely accepted that the excessive activity of myofibroblasts and abnormal collagen deposition is critical for HS formation. Thus, the expression patterns of α‐SMA and Collagen I were also detected in the clinical samples. As expected, at both messenger RNA (mRNA) and protein levels, α‐SMA and Collagen I expressions were significantly upregulated in HS tissues compared with NS tissues (Figure 1A,B). Interestingly, after analyzing the correlation between the expressions of PD‐L1 with α‐SMA and Collagen I on HS tissue samples, it was shown that PD‐L1 is positively correlated with both α‐SMA (*r* = .8461, *p* = .0020) and Collagen I (*r* = .8327, *p* = .0028) (Figure 1C).

### 3.2 PD‐L1 is highly expressed in HFBs

**Figure 2:**
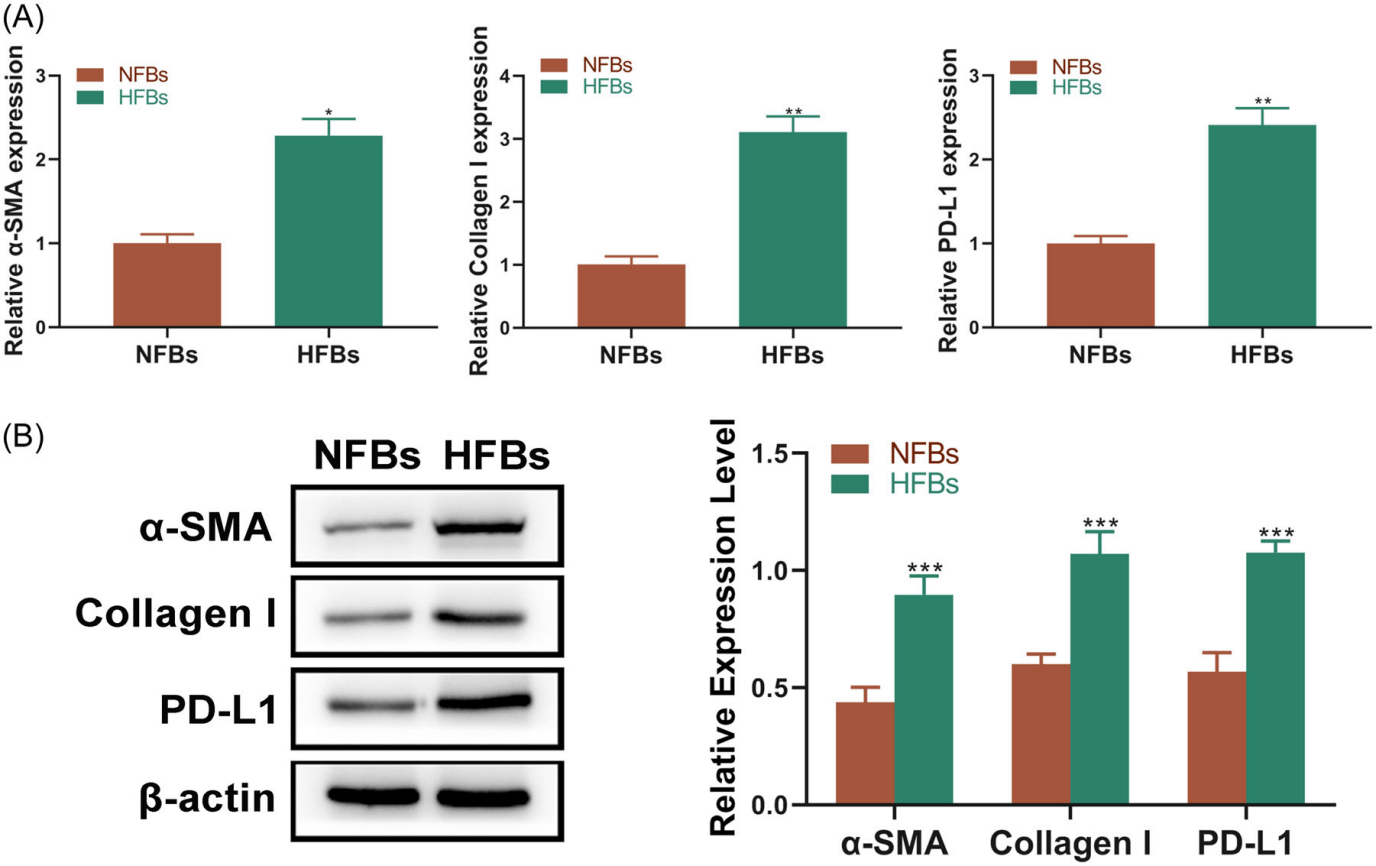
The expression of PD‐L1, α‐SMA, and Collagen I in HFBs is higher than those in NFBs. (A) The mRNA expression level of PD‐L1, α‐SMA, and Collagen I between NFBs and HFBs was analyzed by qRT‐PCR (*n* = 3). (B) The protein expression level of PD‐L1, α‐SMA, and Collagen I between NFBs and HFBs was analyzed by western blot (*n* = 3) (*p < .05, **p < .01, and **p < .005 vs. the NFBs group). HFB, fibroblasts derived from HS tissue; mRNA, messenger RNA

The analysis on fibroblasts derived from HS and NS tissues (HFBs and NFBs) exhibited a similar result that both the mRNA and protein expression levels of PD‐L1 in HFBs were significantly higher than those in NFBs (Figure 2A,B). Meanwhile, qRT‐PCR analysis showed that α‐SMA and Collagen I were highly expressed in HFBs compared with NFBs (Figure 2A), which was further confirmed by western blot (Figure 2B). Combined with the analysis on tissues, our results suggest that the upregulation of PD‐L1 may play a role in HS formation.

### 3.3 BMS‐202 suppressed the cell proliferation, migration, as well as collagen synthesis of HFBs

**Figure 3:**
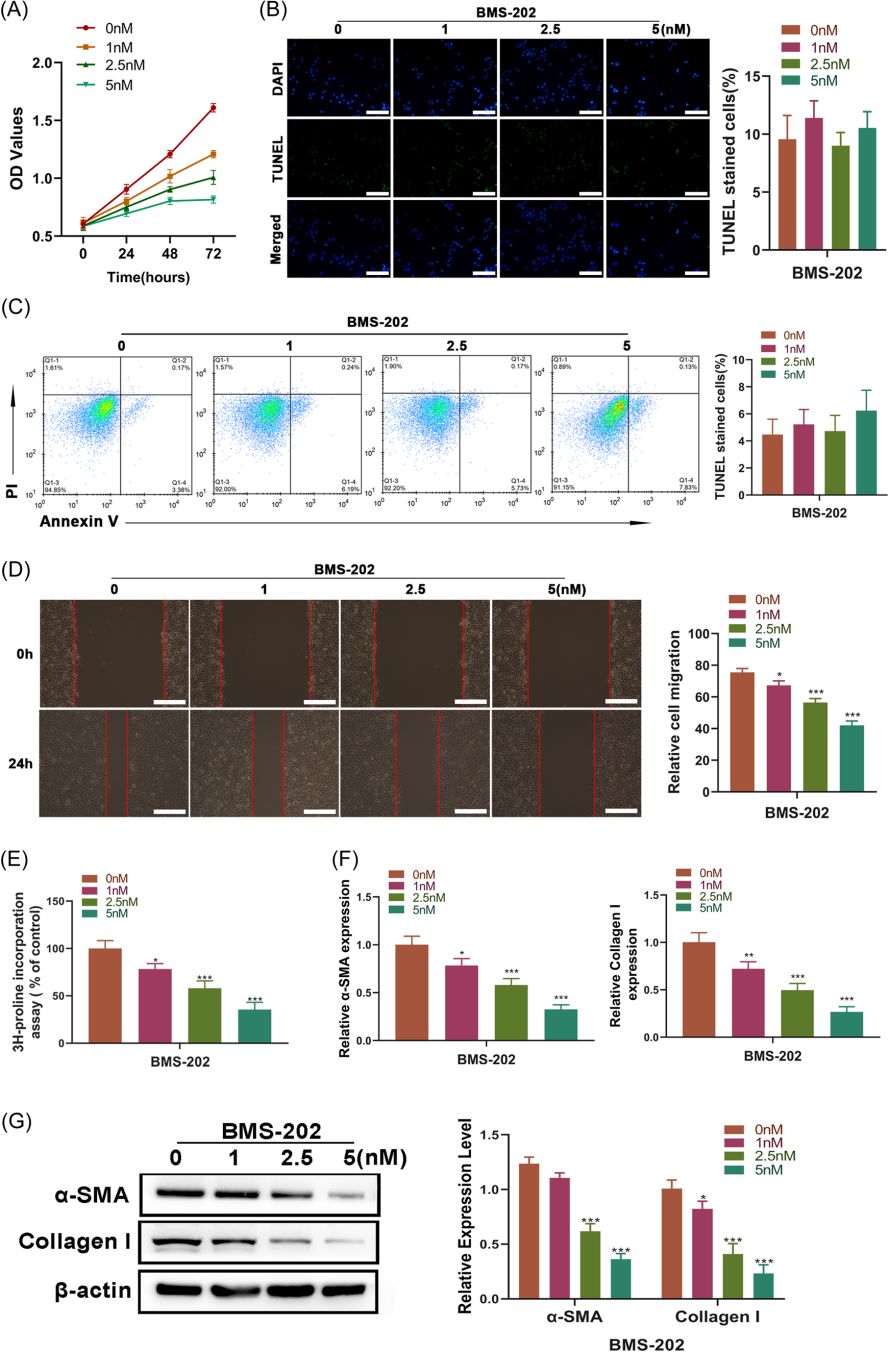
BMS‐202 suppressed the cell proliferaion, migration, as well as collagen synthesis of HFBs. Before subjecting to a series of analyses, HFBs were treated with 0, 1, 2.5, and 5 nM BMS‐202 for 24 h, respectively. (A) The cell viability of HFBs was determined using a CCK‐8 kit after the treatment for 24, 48, and 72 h (*n* = 3). The apoptosis of HFBs was detected by (B) TUNEL and (C) Annexin V/PI staining assays (*n* = 3). (D) The migratory ability of HFBs was detected by wound healing assay (*n* = 3). (E) Collagen synthesis of HFBs was determined by ^3^H‐proline incorporation assay (*n* = 3). (F) The mRNA expression levels of α‐SMA, and Collagen I in HFBs was measured by qRT‐PCR (*n* = 3). (G) The protein expression levels of α‐SMA, and Collagen I in HFBs was measured by western blot (*n* = 3) (*p < .05, **p < .01, and **p < .005 vs. the 0 nM group). CCK‐8, Cell Counting Kit‐8; HFB, fibroblasts derived from HS tissue; mRNA, messenger RNA; TUNEL, terminal deoxynucleotidyl transferase (TdT) dUTP Nick‐End labeling

Since the above result showing the upregulation of PD‐L1 may be related to HS formation, we wonder whether suppressing PD‐L1 could be a novel strategy for HS therapy. To explore the potential of BMS‐202, an inhibitor of PD‐L1, in HS therapy, the effect of BMS‐202 on the cell viability, apoptosis, migration, as well as collagen synthesis of HFBs was subsequently investigated. After treating with different concentrations of BMS‐202 for 24, 48, and 72 h, the cell viability of HFBs from all groups increased in a time‐dependent way, while the proliferative rate of HFBs was restricted by BMS‐202 treatment in a dose‐dependent way (interaction [*F*_(9, 32)_ = 41.53, *p* < .0001]; time [*F*_(3, 32)_ = 491.4, *p* < .0001]; doses [*F*_(3, 32)_ = 194.9, *p* < .0001]) (Figure 3A); however, the apoptosis of HFBs displayed no significant change after treatment (Figure 3B,C). Besides, wound healing and [^3^H]‑proline incorporation assays showed that the treatment of BMS‐202 could suppress not only the migratory ability but also the collagen synthesis capacity of HFBs (Figure 3D,E). Notably, the effect of BMS‐202 on the cell migration and collagen synthesis of HFBs also exhibited a concentration‐dependent way. Furthermore, our results showed that BMS‐202 also caused a significant decrease in both α‐SMA and Collagen I expression at mRNA and protein levels (Figure 3E,F), suggesting that BMS‐202 has the potential of suppressing ECM deposition. Taken together, these data suggested that BMS‐202 could repress cell proliferation, migration, as well as collagen synthesis of HFBs, but has no effect on the apoptosis of HFBs.

### 3.4 BMS‐202 suppressed the ERK and TGFβ1/Smad signaling pathways in HFBs

**Figure 4:**
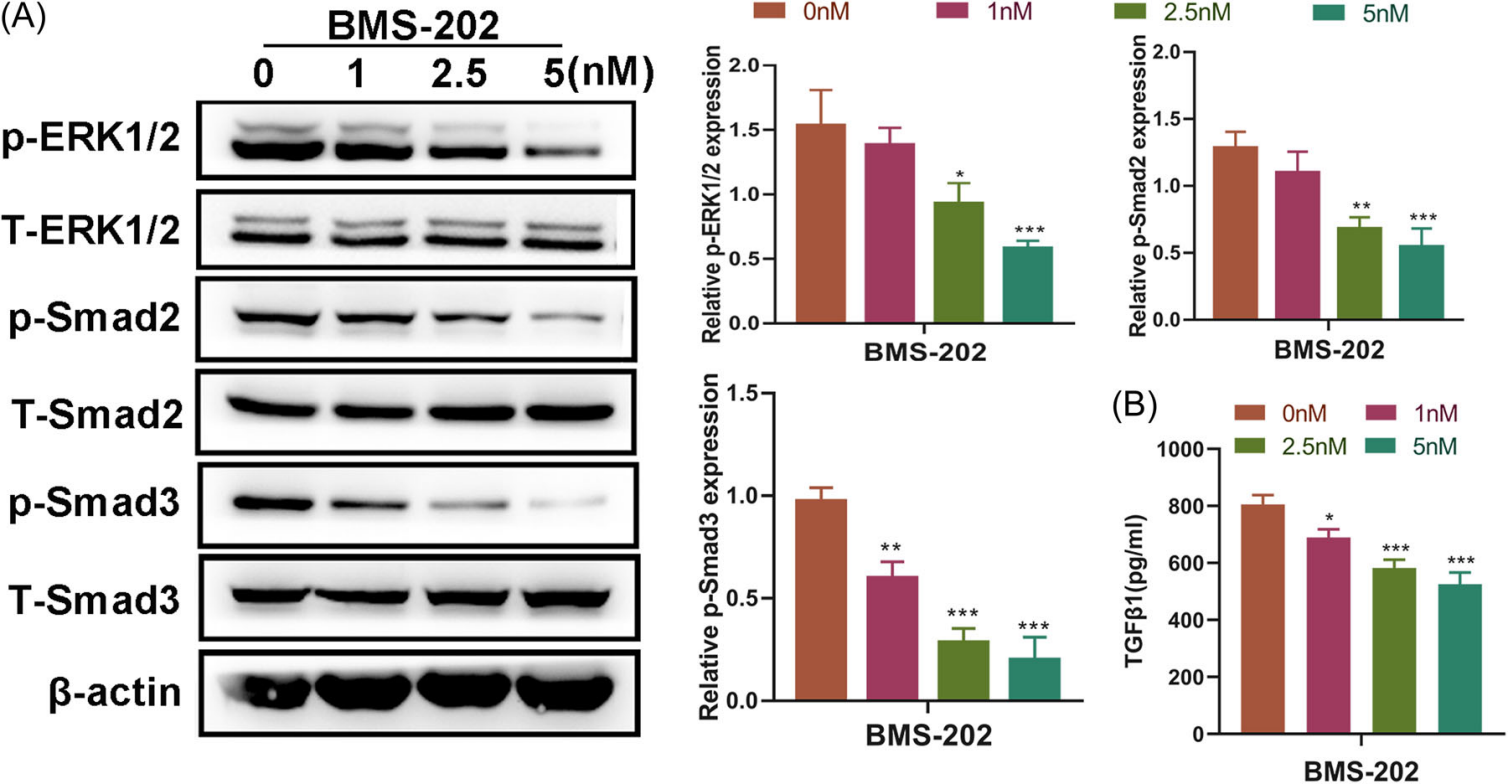
BMS‐202 downregulated the ERK and TGFβ1/Smad signaling pathways in HFBs. (A) Western blot was performed to detect the expression of p‐ERK1/2, T‐ERK1/2, p‐Smad2 (S467), T‐Smad2, p‐Smad3 (S423/S425), and T‐Smad3, in HFBs treated with different concentrations (0, 1, 2.5, and 5 nM) of BMS‐202 (*n* = 3). (B) The expression levels of TGFβ1 in HFBs treated with different concentrations of BMS‐202 was detected by ELISA (*n* = 3) (*p < .05, **p < .01, and **p < .005 vs. the 0 nM group). ELISA, enzyme‐linked immunosorbent assay; HFB, fibroblasts derived from HS tissue; TGFβ1, transforming growth factor beta 1

To further investigate the molecular basis of BMS‐202 in repressing HS formation, we detected the effect of BMS‐202 on the ERK and TGFβ1/Smad signaling. It was observed that Smad3 phosphorylation was significantly inhibited by BMS‐202 in a concentration‐dependent way, whereas the phosphorylation of ERK1/2 and Smad2 were not affected when the concentration of BMS‐202 was less than 2.5 nM (Figure 4A). Meanwhile, TGFβ1 expression was suppressed as the BMS‐202 increasing (Figure 4B). These data revealed that BMS‐202 simultaneously suppressed the ERK and TGFβ1/Smad signaling pathways in HFBs once its concentration reached 2.5 nM in HFBs.

During HS formation, fibroblasts share many malignant phenotypes of cancer cells, including excessive proliferation, atypical differentiation, as well as apoptosis resistance.^21^, ^22^ In the past two decades, immune checkpoint inhibitors, particularly those targeting PD‐1/PD‐L1, provide significant clinical benefits for patients across cancer types.^23^ Increasing evidence supported that the accumulation of fibroblasts is closely related to the immune reactions in fibrotic or malignant lesions.^24^, ^25^ Recently, PD‐L1 has been identified in fibroblasts of lung fibrosis, which possibly contributes to invasion and fibrosis in idiopathic pulmonary fibrosis.^26^ During HS formation, the expression of TGFβ is increased and plays a role in collagen synthesis and deposition, the differentiation of myofibroblasts, as well as ECM deposition.^27^ Jung et al.^28^ demonstrated that TGFβ upregulates the expression of PD‐L1 in both human and murine fibroblasts, and silencing PD‐L1 attenuated the TGFβ‐dependent induction of ECM deposition and cell migration. Based on previously published studies, the present study preliminarily explored the potential role and underlying mechanism of PD‐L1 inhibitor on the treatment of HS.

Initially, our data showed that the expression of PD‐L1 in HS tissues and HFBs was respectively higher than that in NS tissues and NFBs. In the analysis of clinical samples, we also observed there is a positive correlation between the expression of PD‐L1 with both α‐SMA and Collagen I. That's means PD‐L1 may be involved in the development of HS. BMS‐202, one of the most potent small‐molecule inhibitors of PD‐1/PD‐L1, is developed by Bristol‐Myers Squibb, which could strongly bind with PD‐L1 to induce the dimerization PD‐L1 thereby suppressing the function of PD‐L1.^28^ It has demonstrated that BMS‐202 exerted suppressive function on several tumors in recent years.^16^, ^17^ During the reparation of trauma, fibroblasts exhibit aberrant function and activity, which are considered the main active and structural cells in the formation of HS. Thus, suppressing fibroblast activation, proliferation, migration, collagen synthesis, and inducing apoptosis is an important strategy for the treatment of HS. To explore the biological function of BMS‐202 on the pathological fibroblasts, HFBs derived from patients with HS were administrated with a series of concentrations of BMS‐202.

The enhanced proliferation of HFBs is the primary characteristic of HS,^29^ our study revealed that BMS‐202 could effectively impair the cell proliferation of HFBs. The enhanced migration and apoptosis resistance are other pathological cellular behaviors of HFBs. Here, BMS‐202 had a significant dose‐dependent inhibitory effect on the migration of HFBs, while it is not effective on apoptosis. It's widely accepted that superabundant deposition of ECM, which is mainly composed of collagen, is a considerable feature in HS formation.^30^ The capacity of the drug on collagen synthesis is one of the considerable factors to evaluate its potential for HS treatment.^31^ Our study assessed the collagen synthesis with [^3^H]‑proline incorporation assay and the result showed that the treatment of BMS‐202 caused a significant decrease in collagen synthesis of HFBs. Consistent with previous studies, the visibly enhanced expression of collagen I and α‐SMA in HS were investigated in this study. We further investigate the effect of BMS‐202 on the expression of α‐SMA and collagen I in HFBs due to their important functions during the formation of fibrotic ECM environment.^2^ In our study, a decrease in α‐SMA and collagen I expression was expected with the BMS‐202 treatment. These results indicated that BMS‐202 could suppress HFBs proliferation, migration, and collagen synthesis, hinting that BMS‐202 possesses a therapeutic potential against HS.

The central role of TGF‐β1 signaling in the development of HS is widely accepted.^32^ This pathway mainly includes Smad2/3, Ras/MEK/ERK, ETS‐1, etc., among which Smad2/3 pathway is a major signaling pathway that causes the formation of HS.^33^ Smads are comprised of three domains: the amino (N) terminal phosphorylation domain, the central linker region domain, and the carboxy (C) terminal phosphorylation domain that binds to TGFBRI.^34^ In the process of tissue fibrosis, TGF‐β1 binds TGFBRII, which binds TGFBRI, which phosphorylates Smad2/3, which bind Smad 4 and translocate to the nucleus and bind Smad binding elements in gene promoters.^35^ A growing body of research demonstrated that inhibiting the TGF‐β1/Smad2/3 pathway could efficiently reverse the pathological phenotypes of HFBs,^36^, ^37^ which is consistent with our research. Our study revealed that BMS‐202 attenuated pathological phenotypes of HFBs by regulating canonical TGF‐β1 signaling pathway as the C terminal phosphorylation of Smad2/3 in HFBs was significantly suppressed after BMS‐202 treatment. ERK has been reported to phosphorylate Smad2/3 linker region thereby regulating the biological function of Smad2/3 in fibroblasts.^38^, ^39^ Hough et al. demonstrated that inhibiting ERK signaling significantly repressed the phosphorylation of Smad linker region.^40^ Interestingly, our data showed that BMS‐202 treatment reduced the phosphorylation levels of ERK1/2, suggesting a suppressive role of BMS‐202 in ERK pathway. These results revealed that BMS‐202 could reduce the TGF‐β1 expression and the phosphorylation of Smad2/3 and ERK in HFBs, which suggested that BMS‐202 suppressed HFBs proliferation, migration, and collagen synthesis via the ERK and TGF‐β1/Smad pathways.

However, it should be acknowledged there are several limitations in this study. First, the present study only used fibroblasts cultured in vitro on tissue culture material, which is not the same as fibroblast activity in vivo (fibroblasts lose some of their properties as they adapt to tissue culture), which lends some limitation to the translatability of our findings. Besides, there are also many other pathways related to TGF‐β1 signaling and/or fibroblast activation we did not explore, such as p38 MAPK, MRTF/SRF, YAP/TAZ, etc. In addition, further in vivo experiment is necessary to verify the potential therapeutic value of BMS‐202 in HS treatment.

In conclusion, this study confirmed that PD‐L1 was upregulated in HS tissues and derived fibroblasts, which was positively correlated with α‐SMA and Collagen I. Our study demonstrated that BMS‐202, a potent inhibitor of PD‐L1, effectively inhibits the proliferation, migration, and collagen synthesis of HFBs, as well as the expression of α‐SMA and Collagen I. Also, this study suggested that BMS‐202 reverse the pathological phenotypes of HFBs via TGF‐β1/Smad and ERK pathways. Together, this study suggests the potential therapeutic value of BMS‐202 on HS in future clinical applications.

## AUTHOR CONTRIBUTIONS

**Yuanyuan Cai, Min Xiao, and Tianlan Zhao**: designed the research plan. **Yuanyuan Cai, Min Xiao, Xinqing Li, Shanyu Zhou, Yangyang Sun, Wenyuan Yu, and Tianlan Zhao**: performed the experiments. **Yuanyuan Cai and Tianlan Zhao**: wrote the manuscript. All authors have read and approved the final manuscript.

## CONFLICTS OF INTEREST

The authors declare no conflicts of interest.

## ETHICS STATEMENT

All enrolled patients signed written informed consent. This study was reviewed and approved by the Ethics Committee of the Second Affiliated Hospital of Soochow University.

## Data Availability

All data generated or used during the study appear in the submitted article.
